# Perplexing Case of Wilkie's Syndrome: A Rare Presentation in a Young Patient

**DOI:** 10.7759/cureus.5085

**Published:** 2019-07-05

**Authors:** Syed Hamza Bin Waqar, Anosh Aslam Khan, Osama Mohiuddin

**Affiliations:** 1 Internal Medicine, Civil Hospital Karachi, Dow University of Health Sciences, Karachi, PAK; 2 Internal Medicine, Dow University of Health Sciences, Karachi, PAK

**Keywords:** wilkie's syndrome, sma, duodenum, compression, bowel obstruction, postprandial pain

## Abstract

Wilkie's syndrome, also commonly known as superior mesenteric artery (SMA) syndrome, is an infrequent and rare cause of small bowel obstruction. It is caused by extrinsic compression of the duodenal segment of the intestine between the aorta and SMA, causing significant post-prandial abdominal pain and vomiting. The literature suggests the incidence of 0.01% to 0.3%. We present here an atypical presentation of SMA syndrome in which a young patient presented to our tertiary set-up with unusually constant abdominal pain and weight loss requiring invasive interventions.

## Introduction

A proximal intestinal obstruction can pose diagnostic challenges to physicians because of the intricacy of the gastrointestinal anatomy. Superior mesenteric artery (SMA) syndrome is an unusual presentation of a gastrointestinal obstruction leading to an array of symptoms. It was first described in 1861 and since then, case reports have been documented but still, the prevalence remains well below 0.3%. It is caused by the loss of mesenteric fat in between the aorta and the SMA, which results in compression of the third part of the duodenum [1-2]. Here, we present a case of a 19-year-old female who presented to our hospital with significant weight loss and excruciating abdominal pain with vomiting. She was later diagnosed with SMA syndrome and was treated with non-surgical intervention.

## Case presentation

A 19-year-old female, a diagnosed case of atypical bilateral trigeminal neuralgia, primary Raynaud's phenomenon, and endometriosis presented to our tertiary care setup after being referred for intractable nausea and vomiting with excruciating abdominal pain. Initially, these symptoms seem to have started when she had episodes of trigeminal neuralgia, which started three years back, however, all of her symptoms have progressed and occurred out of the window of neuralgia. Vomiting, in particular, worsened after microvascular decompression surgery for atypical trigeminal neuralgia, two years before presentation to our hospital. She had 25 episodes of forceful, projectile, watery, nonmucoid, and bilious vomiting with associated nausea and shortness of breath. She also had some blood streaks in her vomitus on hospital admission. It peculiarly aggravated on eating and relieved to a slighter extent on fasting. Her normal baseline for vomiting was six episodes per day. During such episodes, she felt as if she was going to die. She did not feel nauseous in between the episodes. Her abdominal pain was constant and mainly located in the epigastrium and left upper quadrant. It was a sharp, non-radiating, and lancinating pain with a severity of nine on the standard numeric pain scale. It got worse on eating food and even by drinking water. The pain was not relieved on lying prone, in the left lateral decubitus, or in the knee-chest position. She also felt bloated all the time but had no change in bowel habits. The abdominal pain and vomiting were trialed with amitriptyline, metoclopramide, pregabalin, ketorolac, sucralfate, ranitidine, omeprazole, and antacids, but none were successful. Her weight dropped from 154 pounds to 110 pounds within six months before presentation. There was no associated fever, vertigo, nystagmus, night sweats, dysphagia, melena, or feculent material in the vomitus.

On admission, she looked frail, wasted, and in significant discomfort but was alert and fully oriented with no mood alterations. The patient was afebrile, with a pulse of 102 beats per minute (bpm), blood pressure of 103/62 mmHg, and respiratory rate of 20 per min. Her abdomen was scaphoid, tender to light and deep palpation over the epigastrium and left upper quadrant. Bowel sounds were normoactive in all four quadrants with no signs of hepatosplenomegaly. The patient has no lymphadenopathy, edema, cyanosis, clubbing, oral rashes, or thrush. There were, however, severe conjunctival pallor and delayed capillary refill. The cardiovascular system was significant for early systolic murmur in the pulmonic area, signifying an innocent murmur secondary to anemia. The respiratory and nervous system were normal on physical examination.

The complete blood count (CBC) showed a total leukocyte count (TLC) of 2.6 x 10^9^/L, with neutrophils being 40.2% and lymphocytes being 50.3%. Platelets were 183 x 10^9^/L. Red blood cell (RBC) count was 3.07 x 10^6^/ mL (normal: 4.0-5.0 x 10^6^/ mL) with low hemoglobin and hematocrit of 7.1 g/dl and 23%, respectively. The erythrocyte sedimentation rate and C reactive protein levels were within normal limits. The coagulation profile was normal as well. The electrolyte panel indicated a sodium level of 137 mEq/L (normal: 136-146 mEq/L), chloride of 106 mEq/L (normal: 94-107 mEq/L), and potassium of 3.1 mEq/L (normal: 3.5-5.0 mEq/L). The glycosylated hemoglobin (HbA1c) level was 4.2%, with a random blood sugar level of 97 mg/dl (normal: 79-160 mg/dl). Total serum protein was 5.2 g/dl (normal: 6-8.3 g/dl). The blood urea nitrogen and creatinine ratio was marginally elevated.

**Figure 1:**
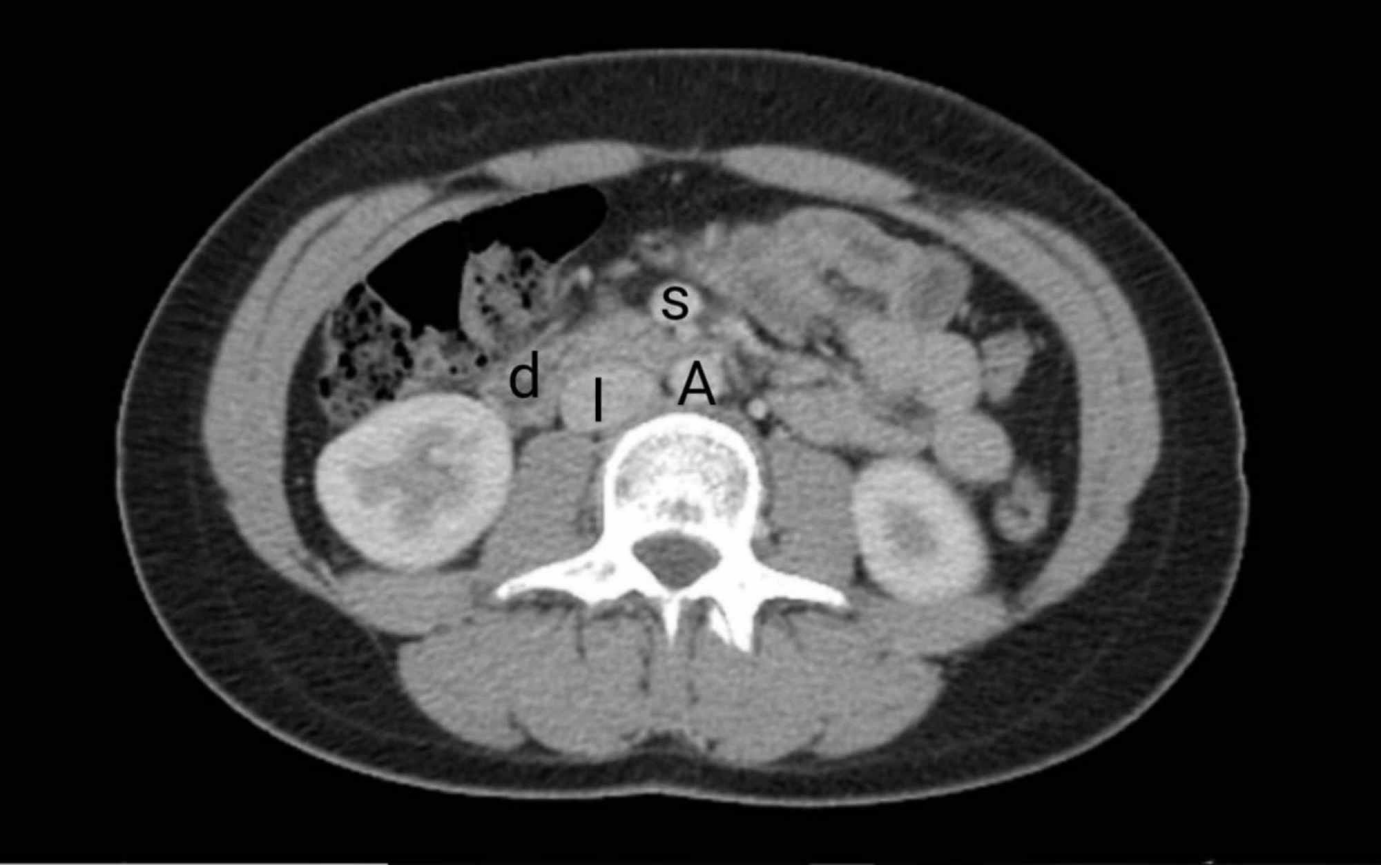
Computed tomography (CT) of the abdomen with contrast showing extrinsic compression of the duodenal segment (d) in between the aorta (A) and superior mesenteric artery (s) I: inferior vena cava.

**Figure 2:**
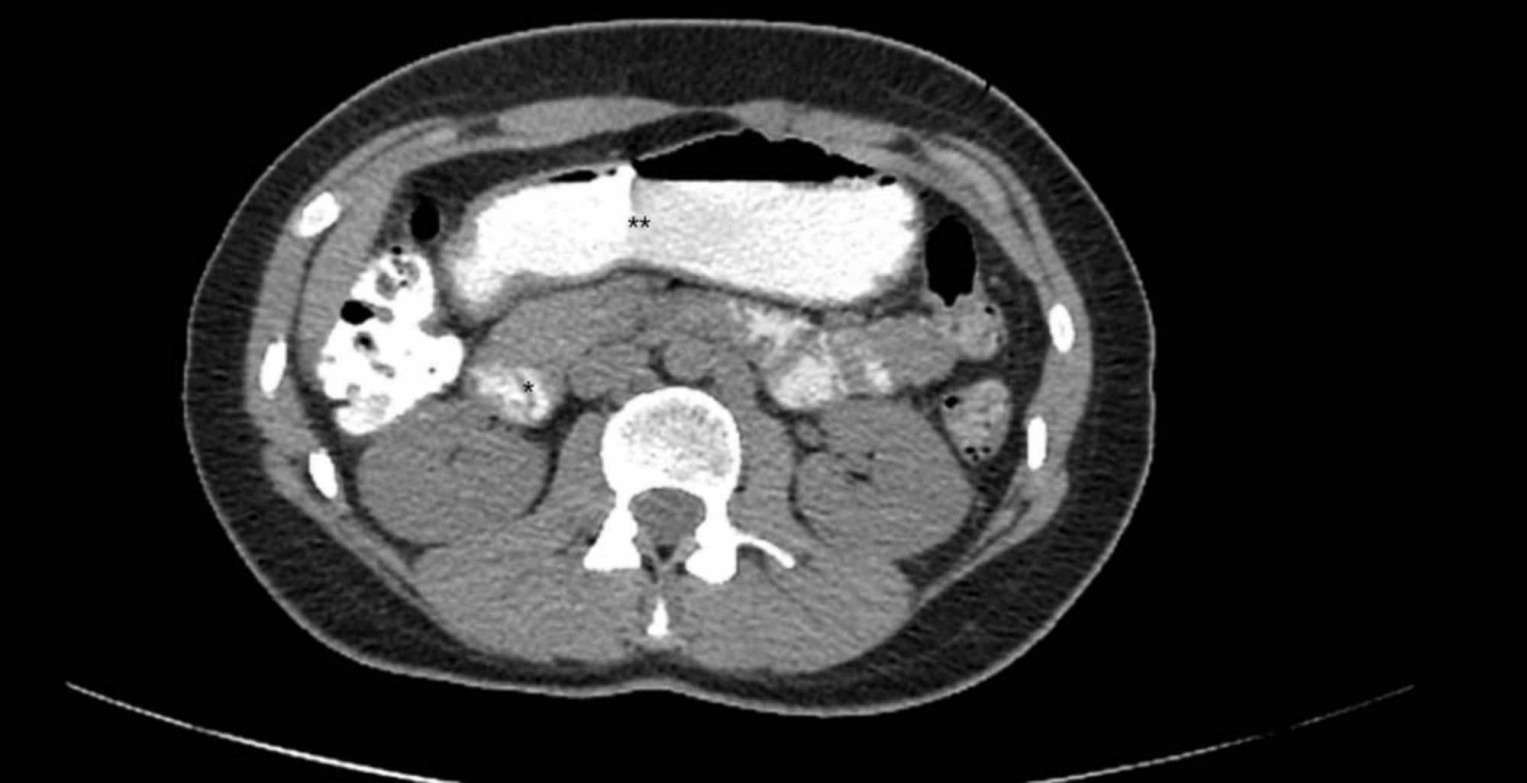
Computed tomography (CT) of the abdomen with oral contrast showing gastric distension (**) and duodenal compression with retention of contrast (*)

Non-invasive imaging was done, which included abdominal ultrasound and plain supine abdominal radiograph (AXR). The ultrasound was insignificant while AXR showed gastric distension. Gastric emptying studies with barium swallow were initially not conducted, as the patient was not able to co-operate because of an aversion to liquids. Esophagogastroduodenoscopy (EGD) was performed, which showed mild erythema consistent with reflux and retained gastric content in the stomach, findings suggestive of gastroparesis. Computed tomography (CT) with oral contrast and intravenous (IV) contrast was performed. Axial and sagittal sections were obtained, which showed gastric distension with extrinsic compression of the third part of the duodenum by the SMA, with retained fluid, consistent with findings of SMA syndrome (Figures 1-2).

**Figure 3:**
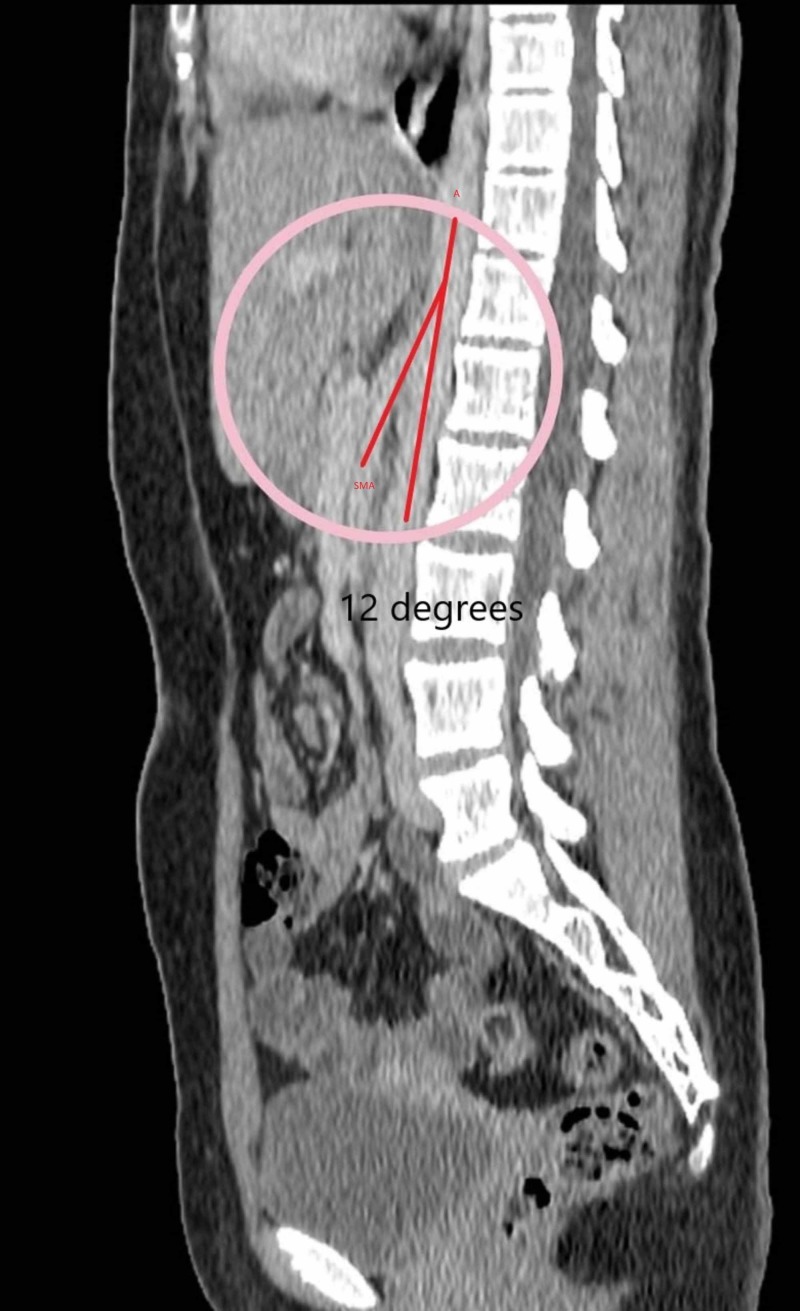
Computed tomography (CT) sagittal view showing aortomesenteric angle of 12 degrees by two red lines drawn over the aortic axis (A) and the superior mesenteric artery (SMA) at the origin (inscribed in a circle)

CT sagittal view showed reduced aortomesenteric distance and angle of about 3.4 mm (normal: 10-28 mm) and 12 degrees (normal: 38-65 degrees) respectively (Figure 3).

Imaging was obtained with the help of the granisetron patch to keep the patient from any emetic episode while imaging.

Supportive and medical treatment was initiated with close liaison between gastroenterologists and nutritionists. To counter the drastic weight loss, the patient was started on total parenteral nutrition (TPN) via a peripherally inserted central catheter (PICC), with dextrose 8%, amino acids 4.3%, sodium acetate 110 mEq at 84 ml/hr and fat emulsion 20% at 6 ml/hr for 24 hours. This diverted the nutritional flow from the gut and helped in pain relief. Nasogastric decompression could not be performed, as it would have aggravated trigeminal neuralgia. Electrolytes were corrected and a jejunostomy feeding tube was used as a possible intervention to improve the metabolism of the patient. She was then followed up for nine months and showed massive improvement in her weight and symptoms.

## Discussion

Superior mesenteric artery (SMA) syndrome, also known as Wilkie’s syndrome, is an atypical and unusual cause of a proximal intestinal obstruction that manifests due to extrinsic compression of the third part of the duodenum between aorta and SMA. It was first reported in 1861 by an Austrian professor, Carl von Rokitansky, and details on pathophysiology and diagnostics indications were described by Wilkie in 1927. It has also been referred to as chronic duodenal ileus, arterio-mesenteric duodenal compression syndrome, and cast syndrome [1] Due to the rarity of presentation, the reported incidence of SMA syndrome ranges approximately between 0.01% and 0.3%. It has been observed more commonly among young adults between the first and fourth decades of life, among which females are one and a half times more prone to develop it as compared to males. However, it can subsequently occur at any age. No racial and ethnic predisposition has been identified yet [2].

In terms of embryogenesis, the duodenum is a derivative of the distal foregut and proximal midgut and, therefore, enjoys dual blood supply from the celiac and superior mesenteric artery. During the fifth week of gestation, physiological umbilical herniation of the midgut occurs followed by a 270° counterclockwise rotation around the axis formed by SMA. By the tenth week, reduction of the intestinal coil takes place such that the SMA runs anteriorly to the horizontal portion of the duodenum. At the level of the "L1" vertebra and behind the body of the pancreas, SMA originates from the abdominal aorta. On the lateral view, SMA runs ventrally and caudally from the anterior aspect of the aorta forming an acute downwards angle. In the erect posture of humans, this aortomesenteric angle ranges from 38° to 65° and is maintained by the left renal vein, the uncinate process of the pancreas, retroperitoneal lymphatics, and fat tissue. The normal physiological aortomesenteric distance lies between 10 mm and 28 mm. As the duodenum is suspended by the ligament of Treitz within the angle range, it is prone to get pinched [2-3].

In SMA syndrome, the aortomesenteric angle acutely decreases, ranging from 6 degrees to 16 degrees, which narrows the base of the aortomesenteric triangle to as low as 2 millimeters. This potentially leads to extraluminal entrapment of the duodenum. Often, the left renal vein may also get compressed, resulting in nutcracker syndrome [4]. There are also documented reports of the coexistence of celiac axis compression syndrome with SMA syndrome [5]. Studies have shown that significant weight loss especially body mass index below the third percentile is one of the most prominent causes of its manifestation [6-7]. Moreover, several congenital and acquired factors have been associated with its pathogenesis. Congenital causes are usually anatomic variations, such as short or hypertrophic ligament of Treitz, short mesenteric root, the low origin of SMA, peritoneal adhesions, and Ladd’s bands. Acquired factors include chronic cachexic conditions, such as cerebral palsy; trauma such as extensive burn injury, dietary disorders leading to severe weight loss, such as anorexia nervosa, postoperative states, such as bariatric surgery, local pathology such as mesenteric root neoplasm, and other conditions such as surgical correction of scoliosis, lumbar hyperlordosis, familial cases, and many more [3].

The clinical presentation of Wilkie’s syndrome may appear acutely or progress insidiously depending on the cause and grade of duodenal impingement. The most common presenting complains are abdominal pain, bilious vomiting, and nausea, often compounded with abdominal distention. Other complaints include acid reflux, early satiety, and food intolerance [1-3]. These symptoms are often worsened by food intake and alleviate when patient lay in the knee-chest or lateral decubitus position [2]. Duodenal compression can also be relieved by the Hayes maneuver, in which pressure is applied below the umbilicus in the cephalic direction, which leads to pain relief due to the relief of tension from the small bowel mesentery [3]. On physical examination, findings are usually nonspecific but can include a distended abdomen, tender epigastrium on deep palpation, a succussion splash, and high-pitched bowel sounds [3]. Persistent vomiting causes dehydration and electrolyte imbalance, such as hypokalemia and metabolic alkalosis among patients [7]. On the other hand, nutcracker syndrome, which may also present due to a narrowing of the aortomesenteric angle, manifests as a left varicocele, menstrual abnormalities, left flank pain, hematuria, and fatigue [2].

It is crucial to diagnose SMA syndrome early so as to prevent significant complications like fatalities due to dehydration and electrolyte imbalance, gastric perforation, gastric pneumatosis, and portal venous gas, and an obstructing duodenal bezoar [8-9].

In addition to being a rare pathology, Wilkie’s syndrome often creates a serious diagnostic dilemma for gastroenterologists due to overlapping clinical presentation with other causes of intestinal obstruction such as diabetes mellitus, collagen vascular disease, biliary pancreatitis, malignant tumors, megaduodenum, intestinal malrotation, scleroderma, pseudo-obstruction, paralytic ileus, constipation, and drug reaction [3,10]. Therefore, radiographic studies are employed in suspected cases of SMA syndrome to ascertain the diagnosis. As a general rule of thumb, a characteristic radiological criterion, also known as Haynes’ criteria, was devised to help in making a diagnosis, which is as follows [11]:

a. Dilatation of the duodenum proximal to obstruction with or without gastric distention

b. The abrupt vertical cutoff in the third portion of the duodenum

c. The anti-peristaltic flow of contrast medium proximal to obstruction, producing to and fro movement

d. Delay of four to six hours in gastroduodenojejunal transit time

e. Relief of obstruction on assuming the knee to chest or left lateral position

The plain abdominal film is mostly nonspecific but often reveals dilatation proximal to the duodenum and absence of distal bowel gas. Although barium studies sometimes show an abrupt narrowing in the third part of the duodenum, it is a less sensitive investigation, as it may turn out to be normal. Endoscopy plays an important role to eliminate any intraluminal causes of obstruction and complications of SMA syndrome such as gastric stasis, biliary reflux, and gastric or duodenal ulcers. Ultrasound is used as an easy and noninvasive modality to identify and measure the aortomesenteric angle. However, recently, computed tomography (CT) and magnetic resonance angiography (MRA) have proved to be standard investigation modalities for the measurement of the aortomesenteric angle and distance and the study of related anatomic details, for instance, the site of duodenal compression, local pathologies, and amount of intra-abdominal and retroperitoneal fat [3,12-13]. Ünal B et al. report that an aortomesenteric angle cutoff of 22° revealed sensitivity and specificity of 42.8% and 100%, respectively, while a cutoff of 8 mm of aortomesenteric distance showed 100% sensitivity and specificity for SMA syndrome [13].

The treatment regimen begins with conservative, medical management to provide relief from the symptoms of obstruction. It includes fluid resuscitation, electrolytes repletion, nasogastric tube insertion for gastric decompression, and parenteral or enteral nutrition supplementation to increase body weight. The patient should be monitored acutely, and gradual oral food intake should be initiated once symptoms begin to abate [10-11]. If conservative management fails, various surgical treatments can be approached. Open abdominal surgeries include gastrojejunostomy, Strong procedure (a division of ligament of Treitz), and duodenojejunostomy. Nowadays, the standard operative method is laparoscopic duodenojejunostomy, which has a success rate of about 80% to 100%. Its main advantages include shorter operative time, minimum blood loss, less postoperative pain, low risk of postoperative adhesions, excellent cosmetic outcomes, early recovery of bowel peristalsis, reduced risk of incisional hernia, and shortened hospital stay [3,11- 12]. In a nutshell, surgery should be pursued if there is a prolonged period of severe and recurrent upper gastrointestinal symptoms (at least once per week for more than six months), characteristic diagnostic findings on imaging, refractory response to conservative medical management, and presence of complications [12].

## Conclusions

The diagnosis of SMA syndrome can pose a significant diagnostic challenge to physicians and, once recognized, can be a bit troublesome to treat. Keeping in view the hazardous complications that may unfold because of SMA syndrome and the unpredictable outcomes, it is pivotal to keep vigilant surveillance on the patients by following up after every few of months with a multidisciplinary team comprising a gastroenterologist, radiologists, and a nutritionist so as to ensure a healthy lifestyle for the patient. Moreover, jejunal feeding with close electrolyte monitoring should be encouraged to remit the symptoms of SMA syndrome.
